# Simplified Models to Assess the Mechanical Performance Parameters of Stents

**DOI:** 10.3390/bioengineering11060583

**Published:** 2024-06-07

**Authors:** Juan P. Toledo, Jaime Martínez-Castillo, Diego Cardenas, Enrique Delgado-Alvarado, Marco Osvaldo Vigueras-Zuñiga, Agustín L. Herrera-May

**Affiliations:** 1 Micro and Nanotechnology Research Center, Universidad Veracruzana, Boca del Río 94294, Veracruz, Mexico; juan.toledo@complx.com.mx (J.P.T.); endelgado@uv.mx (E.D.-A.); 2 Facultad de Ingeniería Eléctrica y Electrónica, Universidad Veracruzana, Boca del Río 94294, Veracruz, Mexico; 3 School of Engineering and Sciences, Tecnologico de Monterrey, Zapopan 45138, Jalisco, Mexico; diego.cardenas@tec.mx; 4 Facultad de Ingeniería Mecánica y Ciencias Navales, Universidad Veracruzana, Boca del Río 94294, Veracruz, Mexico; mvigueras@uv.mx; 5 Facultad de Ingeniería de la Construcción y el Hábitat, Universidad Veracruzana, Boca del Río 94294, Veracruz, Mexico

**Keywords:** stent, FEM, simplified model, ischemic heart disease, shell elements, FDA

## Abstract

Ischemic heart disease remains a leading cause of mortality worldwide, which has promoted extensive therapeutic efforts. Stenting has emerged as the primary intervention, particularly among individuals aged 70 years and older. The geometric specifications of stents must align with various mechanical performance criteria outlined by regulatory agencies such as the Food and Drug Administration (FDA). Finite element method (FEM) analysis and computational fluid dynamics (CFD) serve as essential tools to assess the mechanical performance parameters of stents. However, the growing complexity of the numerical models presents significant challenges. Herein, we propose a method to determine the mechanical performance parameters of stents using a simplified FEM model comprising solid and shell elements. In addition, a baseline model of a stent is developed and validated with experimental data, considering parameters such as foreshortening, radial recoil, radial recoil index, and radial stiffness of stents. The results of the simplified FEM model agree well with the baseline model, decreasing up to 80% in computational time. This method can be employed to design stents with specific mechanical performance parameters that satisfy the requirements of each patient.

## 1. Introduction

The main cause of death worldwide by 2020 was associated with heart disease [1]. During 2022 in the USA, 699,659 deaths were related to heart diseases (21.37%) [2]. Wang et al. [3] reported that the global cost of cardiovascular diseases to reach USD 1044 billion in 2030. Khan et al. [4] estimated that ischemic heart disease (IHD) affects approximately 126 million persons, which is close to 1.72% of the population of the world. There are four therapeutic approaches employed to address ischemic heart disease: (a) non-pharmacological measures around lifestyle habits, (b) pharmacological measures such as blood pressure medications, (c) percutaneous coronary intervention that involves placing a vascular endoprosthesis (a stent) in affected heart vessels to alleviate the ischemic condition, and (d) surgical intervention. For surgical intervention in patients aged 70 years and older, the mortality risk outweighs the benefits of this procedure. Consequently, stenting has emerged as the predominant method for treating ischemic heart diseases [5].

Etave et al. [6] reported one of the first finite element method (FEM) simulations of the mechanical performance parameters of stents. These simulations included mechanical performance parameters such as radial elastic recoil, resistance of the stent to external compressive forces, stent foreshortening, and stent coverage area. Furthermore, these simulations regarded the stent with cyclic symmetry and the inflation driven by radial displacement. The finite element method (FEM) simulations of the balloon inflation process are nonlinear due to the plasticity during balloon expansion and the contact interactions between the stent, balloon, and arterial plaque. Also, Liang et al. [7] developed comprehensive 3D models of the stent and the balloon, incorporating basic nonlinear properties for all material elements. However, a limiting factor in the simulation was the representation of the balloon as a planar cylinder without folding.

Initially, simulation interactions between the balloon and stent were investigated with material linearities on the non-essential components like the balloons. Later, when the computer power capacity was increased, the simulations of stents considered more elements and nodes of the FEM models, as well as nonlinearity parameters [8]. The balloon was changed from a hollow cylinder with linear behavior to a fully folded balloon with hyperelastic material properties. Similarly, the plaque and the artery initially were modeled as a smooth occlusion embedded into a cylindrical artery [9,10]. Later, simulations of stents considered complex geometries based on 3D scans from damaged arteries and hyperelastic materials, as well as Neo-Hookean strain energy function, to describe the mechanical behavior of plaque [11]. However, only two mechanical parameters recommended by the Food and Drug Administration (FDA) were investigated during this research [11].

When the simulations of stents using FEM models incorporated more design parameters, these models were used to optimize the geometrical parameters of stents [12]. In 2010, the FDA established guidelines for engineering tests and technical variables to support the safety and effectiveness of intravascular stents and their delivery systems [13]. These guidelines were considered in the following research on the design and optimization of stents. In addition, hemodynamic behavior started to be investigated using computational fluid dynamics (CFD) simulations due to the relationship with neointimal growth under low artery wall shear stress (WSS) [14].

In 2015, several researchers [15,16,17,18,19] reported optimization processes of stents that included FDA parameters. For instance, Bressloff et al. [15] designed a method considering the FDA parameters of stents to study the geometric modifications of their components. This method incorporated a response surface methodology (RSM) based on the Kriging method. Furthermore, Ragkousis et al. [16] presented a virtual design of experiments to understand the effect of the positioning parameter on some mechanical performance parameters of stents [16]. However, the average time for analyzing a single point was 160 h. Next, Tammareddi et al. [17] improved the optimization process of stents by introducing multi-objective robust optimization of coronary stents [17], decreasing the computational time for an average run to 26 h. In 2017, Li et al. [18] investigated an optimization design of stents based on the Kriging surrogate model, focused on the FDA parameters, and assessed the fatigue of stents [18]. Nevertheless, the balloon was modeled using a simplified cylindrical shape. In 2019, Geith et al. [19] incorporated FEM simulations for modeling balloon folding, pleating, and stent crimping. They compared their results of FEM simulation with experimental results. They only focused on comparing the balloon-stent without considering the FDA parameters. In 2021, Gharleghi et al. [20] examined stent geometries using multi-objective optimization with seven design variables. Furthermore, they used mechanical performance parameters of the stent recommended by the FDA to assess its optimal design. However, the structural analysis of the stent was simplified in terms of the geometry of the balloon and artery using ideal cylindrical shapes.

Herein, we propose a simple method based on FEM models of stents to predict their mechanical performance parameters considering the FDA guidelines. In addition, this method includes the complexities of the balloon folding reported by Geith et al. [19] and Gharleghi et al. [20] but with a reduced computational time. The results of our baseline stent model agree well with the experimental data. Also, our simplified FEM models of stents can determine their main mechanical performance parameters with a reduction in computational time of up to 80%. The proposed method can be employed to design better structures of the stents that comply with the specific requirements of potential patients.

## 2. Materials and Methods

This section describes the stages of the proposed method for evaluating the mechanical performance parameters of stents, considering the FDA guidelines.

**Figure 1:**
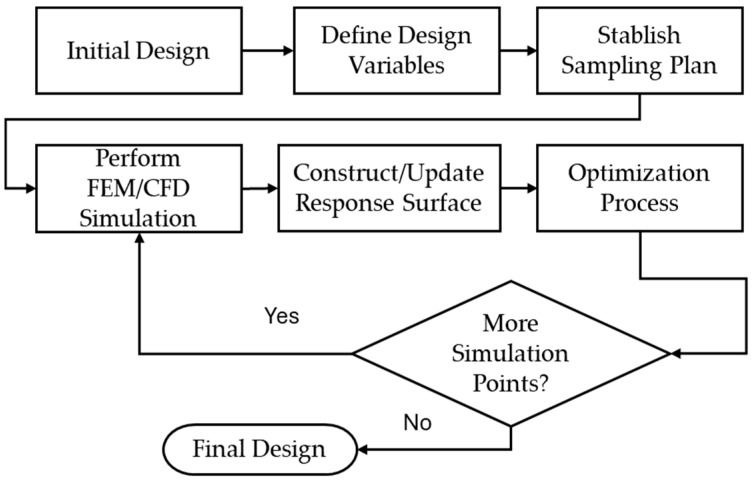
Different stages of the proposed method to assess the mechanical performance parameters of stents.

Figure 1 depicts the different stages of the global optimization process used to predict the mechanical performance parameters of stents using FEM and CFD simulations. From these stages, the stage that requires more computational time is the FEM/CFD simulation.

GSE-Biomedical^®^ (Hermosillo, Mexico) [21] provided the stent’s geometry and experimental results. Two methods were explored. The first method uses a reduced model of the stent with solid elements, while the second employs a reduced model with shell elements. Performance parameters of stents based on the FDA were obtained for all the simulations. Both methods were compared with the information obtained from the simulations reported in the literature.

### 2.1. Mechanical Performance Parameters of Stents

The mechanical performance parameters of stents considering the FDA guidelines are as follows:

1. Percent surface area: The area over which a stent contacts a vessel may affect the biological response of the vessel. The amount of open, non-contact area may influence tissue prolapse or ingrowth. The percentage of area is calculated as 100·(area in contact with vessel/full cylindrical surface area).
2. Foreshortening: Dimensional changes may occur when deploying a stent, influencing the final length. Knowledge of the foreshortening characteristics aids in proper stent length selection and proper placement in the body. Percent foreshortening is determined as 100·(change in length/loaded length).
3. Recoil for balloon expandable stents: The recoil behavior of balloon expandable stents influences proper device selection, sizing, acute post-implant results, and long-term clinical outcomes. Recoil is a function of stent design and material selection. Therefore, knowledge of stent recoil helps to characterize the behavior of a particular stent design. Recoil is calculated as the diameter of the expanded balloon/diameter of the deflated balloon.
4. Radial stiffness and radial strength: Radial stiffness and stent recoil determine the diameter of balloon expandable stents deployed in compliant vessels. Radial stiffness and radial strength characterize the stent’s ability to resist collapse under short-term or long-term external loads. The radial stiffness of the stent is calculated as the force required to compress a length normalized stent/amount of compression. The units of the radial stiffness are N/mm. This radial strength is defined as the force that generates a plastic deformation on the expanded stent. It is computed when the radial stiffness slope decreases. The plastic deformation of the stent began when its radial strength was achieved.

### 2.2. Geometry and FEM for Baseline Stent and Balloon Model

**Figure 2:**
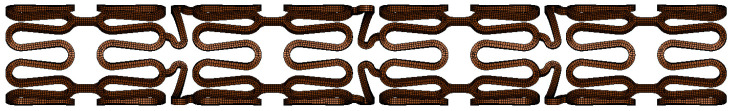
FEM model of the stent provided by GSE-Biomedical^®^ (Hermosillo, Mexico) [21].

The stent device has an inner diameter of 1.16 mm, a thickness of 80 μm, and a total length of 9.416 mm. For this stent, we developed its FEM model using the LS-DYNA software (ANSYS, San Diego, CA, USA), as shown in Figure 2. This model contains three layers of elements along the thickness of the stent, incorporating 42,657 hexahedral solid elements of formulation 2. In LS-DYNA R11.1.0 software, a solid element formulation 2 is a fully integrated selective reduced (S/R) solid element. The computer used for all simulations of the stents was a Dell Precision Workstation T5810 (Round Rock, US), with a processor Intel^®^ Xeon^®^ E5-2697 v4 -18 C; 2.3 GHz, 2400 MHz, 45 MB, 64 GB on RAM. For this study, only 14 processors were used in any given simulation.

**Figure 3:**
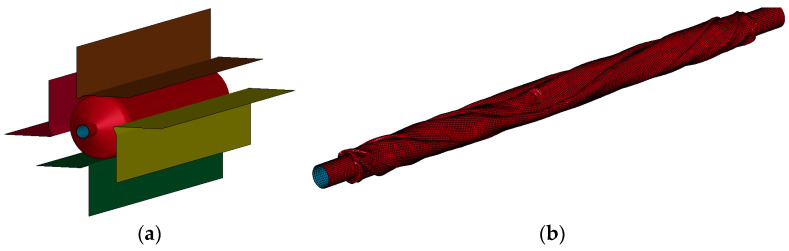

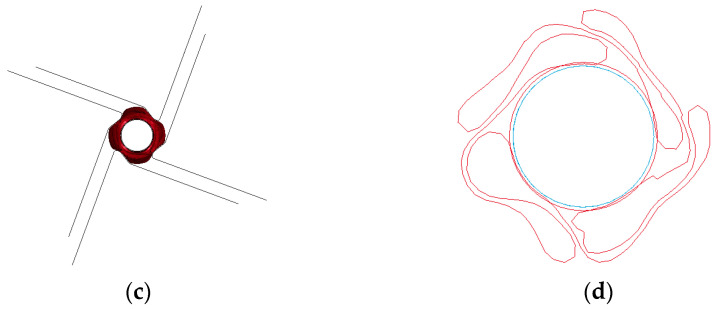
(**a**) The initial stage of the process of balloon pleating, (**b**) an isometric view of the meshed balloon once it is folded, (**c**) a front view of the balloon with its pleating tools, and (**d**) a cross-section of the balloon once the pleating is completed.

The FEM model of the balloon (see Figure 3) was developed following the procedure reported by Geith [19] and Oberhofer [22]. In this procedure, the balloon starts off inflated, and then its pleating is simulated. The expanded balloon has an internal diameter of 3 mm, a thickness of 20 μm, and a total length of 20 mm with an effective length of 15.54 mm. It has an inner stent diameter of 0.63 mm. Furthermore, this FEM model uses shell elements fomulation 1 that are related to the Hughes–Liu element formulation used in the LS-DYNA software. This FEM model of the balloon incorporates 53,938 hexahedral elements with an average dimension of 0.05 mm. Thus, the total number of elements for the FEM model that includes the stent and balloon elements is 96,595.

### 2.3. Materials for Balloon and Stent

**Figure 4:**
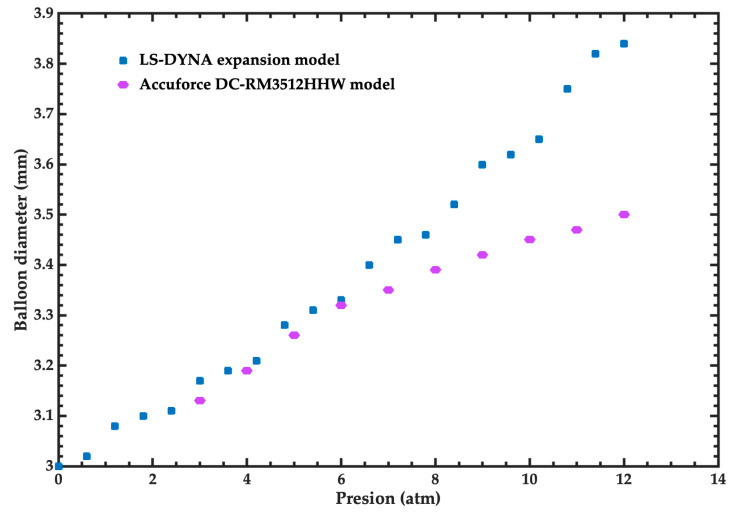
Inflation diameter of the balloon for the Accuforce DC-RM3512HHW model [23] and FEM model as a function of pressure.

CrCo-L605 is the material used for the stent and its properties were provided by GSE-Biomedical (Hermosillo, Mexico) [21]. This model was simulated using Mat_024_PICEWISE_LINEAR_PLATICITY. This material has a Young’s modulus of 233.89 GPa, a density of 7830 kg/m^3^, a Poisson ratio of 0.3, a yield strength of 1117 MPa, and its plastic properties are described in Table 1. On the other hand, the balloon was modeled using a linear material with a Young’s modulus of 400 MPa, a Poisson ratio of 0.3, and a density of 1000 kg/m^3^. The performance of the balloon was taken from a non-compliant material following the Accuforce DC-RM3512HHW model [23]. The comparison of the inflation diameter as a function of pressure is illustrated in Figure 4. In the pressure range from 3 atm to 8 atm, the performance of the FEM model of the balloon agrees well with the Accuforce model [23]. For this pressure range, the maximum error of the FEM model is 2.06%. On the other hand, the minimum and maximum errors of the FEM model are 0.30% and 9.71% for the pressures of 6 atm and 12 atm, respectively.

### 2.4. Geometry and FEM Model of Simplified Stent

**Figure 5:**
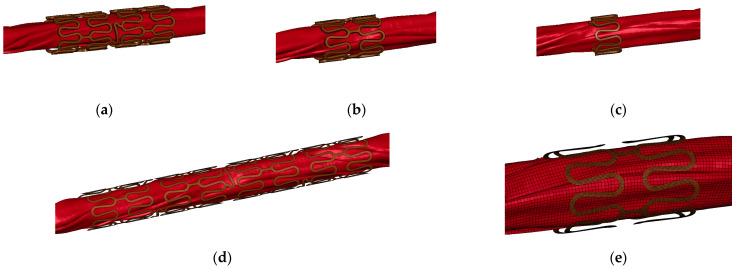
Simplified stent models. Models considering the (**a**) 50% (**b**), 25% (**c**), and 12.5% of the complete stent; models including (**d**) 100% and (**e**) 25% of configuration with shells.

We considered two cases in the FEM model of the stent. The first case employed a solid model using an incomplete stent. This model considered 100% of the balloon dimension and three fractions of the stent (50%, 25%, and 12.5%) based on the symmetry of its geometry. The second case of the FEM model used shell elements instead of solid elements. In this case, we included 100% of the balloon dimension and 25% of the fraction of the stent, as well as a variety of attributes such as integration points and element formulation. The geometries of the different configurations are represented in Figure 5, in which the balloon was cut to better appreciate the configuration of the stent.

Two contact conditions were established on all the models. The first condition was a self-contact condition inside the balloon without a friction coefficient. The second condition was a condition of contact between the balloon and the inner surface of the stent. Based on data published by Gervaso et al. [24] and Pant et al. [25], we performed different simulation iterations until we found that a static friction coefficient of 0.1 and a dynamic coefficient of 0.09 were consistent with the numerical results.

Three configurations were used for the shell elements of the FEM model of the stent. The LS-DYNA element formulation 16 is a fully integrated element; LS-DYNA element formulation 1 is a Hughes–Liu formulation; and LS_DYNA element formulation 2 is a Belytschko–Tsay formulation. In addition, different integration schemes were used along the shell thickness of the FEM model, which were evaluated from 1 to 7 points.

### 2.5. Simulations for Mechanical Performance Parameters of Stent

Two simulations were performed for the baseline and the simplified models. The first simulation consists of the expansion and compression of the balloon in three stages. This simulation was performed for 2 ms and was considered a quasi-static analysis according to Ragkousis et al. [26], in which the dynamic effects were negligible. The first stage consists of the inflation of the balloon with a constant pressure of 6 atm. At the end of this cycle, the average diameter of the balloon is calculated using various measurements at different points of the balloon. In the second stage, the air is sucked from the balloon with twice the vacuum pressure as the first half of the time. At the end of this stage, the outer area of the FEM model was measured to obtain the surface area percent.

**Figure 6:**
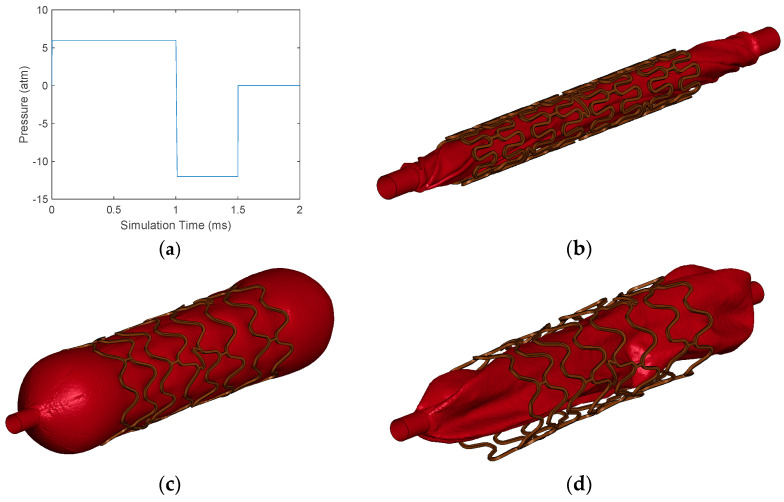
(**a**) Behavior of the pressure applied to the balloon as a function of time. (**b**) The initial state of all the simulations. For this case, baseline simulation is illustrated. (**c**) The final state of the balloon with its geometry fully inflated. (**d**) The final state of the balloon with its geometry completely deflated.

In addition, the length of the FEM model of the stent was determined, and its foreshortening was measured. Furthermore, at the end of the first stage, the final diameter of the stent was estimated, and its recoil was calculated. Figure 6 depicts the different stages of the simulations and the inflation process of the balloon.

**Figure 7:**
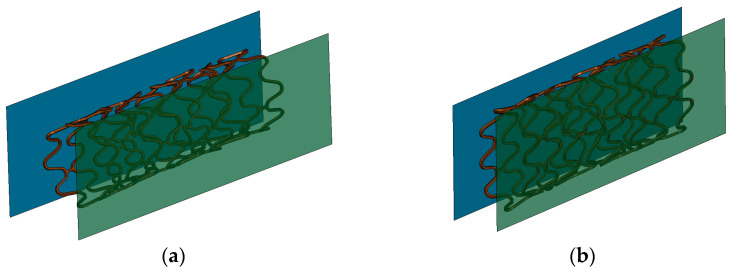
(**a**) Initial configuration and (**b**) final configuration of the compression simulation of the stent.

Once the first simulation is completed, the resulting stent geometry is exported as input for the second simulation. During the second simulation, the final stent geometry is placed between the rigid plates (Figure 7a) and subjected to compression for 1 ms until its final diameter reached 1.8 mm (Figure 7b). A static condition is achieved as in the first simulation. From this simulation, the reaction force of the stent as a function of the wall is obtained, and the radial stiffness as a function of compression can be computed. When the FEM model of the stent begins to show plasticity, its radial strength can be determined.

## 3. Results and Discussion

This section shows the results of the baseline model of the stent in comparison with the experimental data reported by its manufacturer [21]. In addition, the results of the baseline and simplified models of the stents are presented, considering their FDA parameters and simulation times.

### 3.1. Baseline Simulation

**Figure 8:**
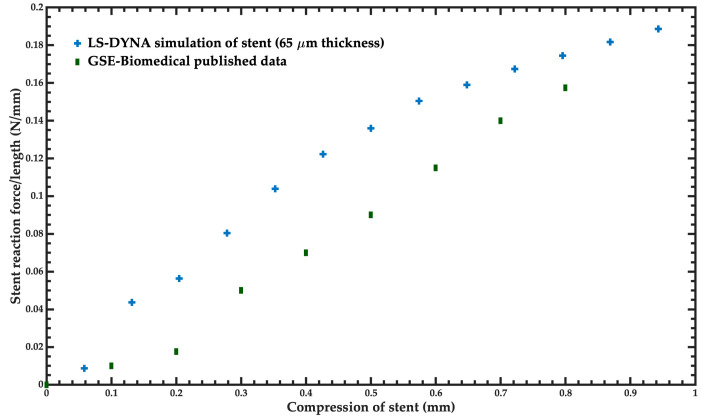
Comparison of the reaction force of the stent under compression, considering the experimental data [28] and the FEM model of the stent.

The results of the baseline model of the stent were compared with experimental data reported by the manufacturer [21,27,28]. These results included the radial recoil, length foreshortening, and radial stiffness of the stent. Figure 8 shows a comparison between the FEM model of the stent and the experimental results for radial stiffness. For this comparison, the thickness of the stent was changed to 65 μm to compare with the final model tested by the manufacturer [28]. In all the following simulations of the stent, we used the original thickness of 80 μm. The slope of the stent reaction force/length of the simulation of the stent has a similar behavior to that of the experimental data. The minimum errors of these simulations are 10.8% and 13.8% for the compressions close to 0.1 mm and 0.8 mm, respectively.

**Figure 9:**
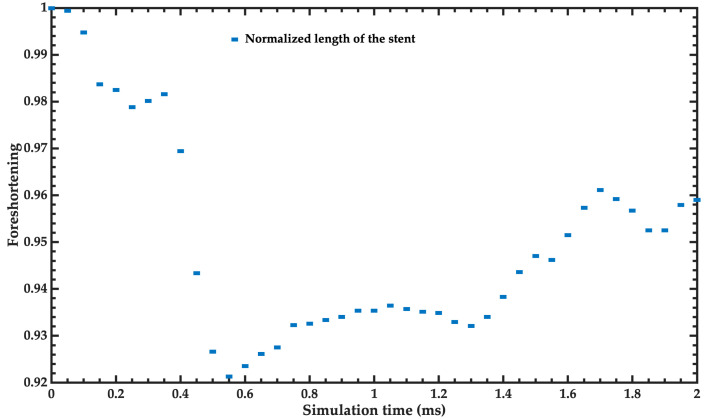
Foreshortening of the stent as a function of the simulation time.

The manufacturer [21] reported a foreshortening of 2%. On the other hand, the foreshortening of the FEM model of the stent is close to 4% at the end of the simulation (Figure 9). The simulation results agree well with the experimental behavior reported by the manufacturer [21,27,28].

**Figure 10:**
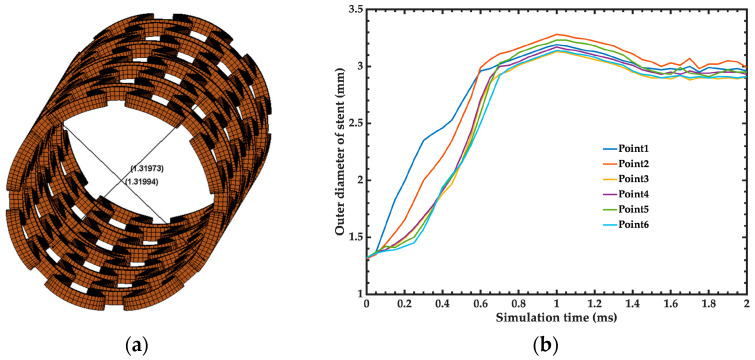
(**a**) Diameter distance at different longitudinal positions were taken to obtain the radial recoil. (**b**) Results of the outer diameter of the stent as a function of simulation time.

The recoil of the stent reported by the manufacturer [21,27,28] is 4.6%, while the recoil obtained by the simulation of the stent is close to 7.8%. Figure 10a depicts the different points of the FEM model of the stent where the measurements were obtained. Figure 10b shows the outer diameters of the stent as a function of the simulation time.

The results of our stent FEM model agree well with the experimental data reported by the manufacturer of the stent [21]. The proposed FEM model may be used to predict the mechanical performance parameters of stents considering the FDA guidelines.

### 3.2. Mechanical Performance Parameters

Once the baseline model of the stent was established and compared with experimental data, we assessed its mechanical performance parameters regarding semicomplete models based on solid and shell elements with different formulations.

First, the length foreshortening of the stent was the first mechanical performance parameter determined.

**Figure 11:**
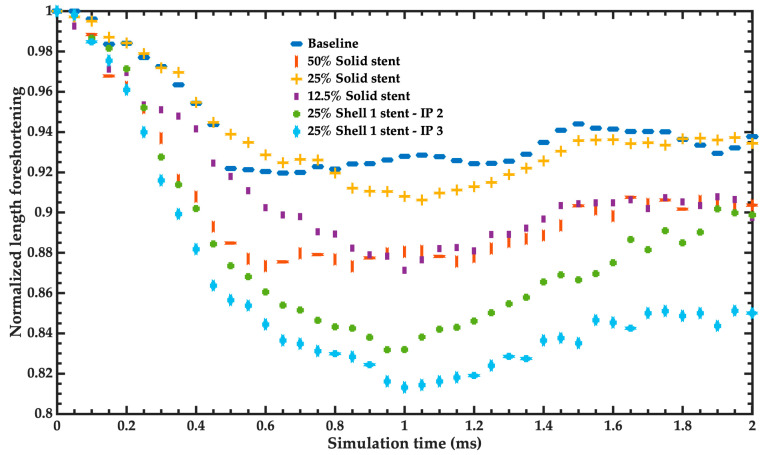
Normalized length foreshortening of the different simplified models in comparison to the baseline model of the stent.

Figure 11 depicts the results of the normalized length foreshortening of the stent as a function of simulation time employing solid or shell elements with different formulations. These results were compared with those of the baseline model of the stent. Regarding error percentage for the last simulation time, the 25% solid model of the stent has an error percentage of foreshortening of 6.57% compared to the baseline model. On the other hand, the 50% solid model and 12.5% solid model of the stent register error percentages of foreshortening of 9.63% and 10.24%, respectively.

The radial recoil of the stent was the second mechanical performance parameter assessed.

**Figure 12:**
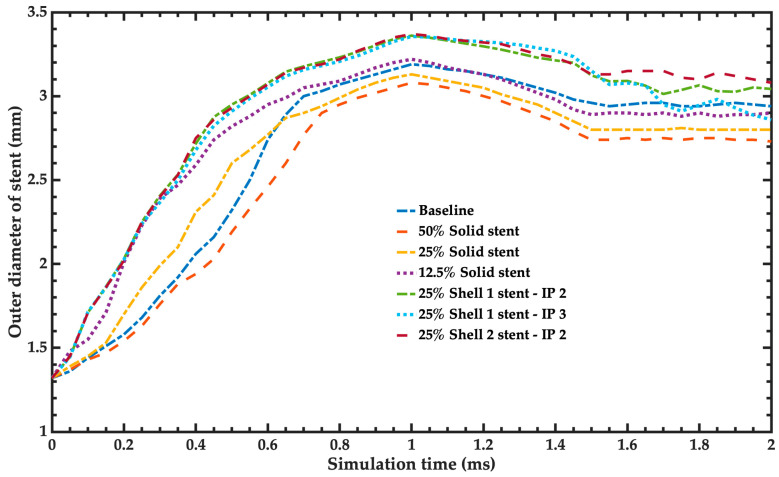
Radial expansion of the balloons of the simplified models and the baseline model. For the shell model, the nomenclature is defined as “ShellXX-IPYY”, where XX represents the formulation used to determine the shell, and YY indicates the number of integration points across the thickness of the element.

Figure 12 compares the radial expansion of the different simplified models and the baseline model of the stent. This comparison helps to identify the simplified model with a behavior closest to the baseline model, considering the same postprocessing time step. The recoil index (*R*) of the stent can define the simulations of the simplified models with better behavior related to the baseline model. For the simplified models of the stents, the number 2 of the recoil index indicates the behavior closest to the baseline model of the stent.

(1) $$R=\frac{D_{1}}{D_{2}}+\frac{D_{3}}{D_{4}}$$

where *D*_1_ is the diameter of the expanded stent of the simplified model, *D*_2_ is the diameter of the expanded stent of the baseline model, *D*_3_ is the diameter of the simplified stent model after the balloon is deflated, and *D*_4_ is the diameter of the baseline stent mode after the balloon is deflated.

Table 2 depicts the results of the recoil and recoil index of the stent simulation models compared with the stent’s baseline model. The data are ordered closer to the baseline model’s recoil index (two values). According to the recoil index, the simplified model that best describes the recoil process is the model of a 12.5% solid stent that considers a solid element.

**Figure 13:**
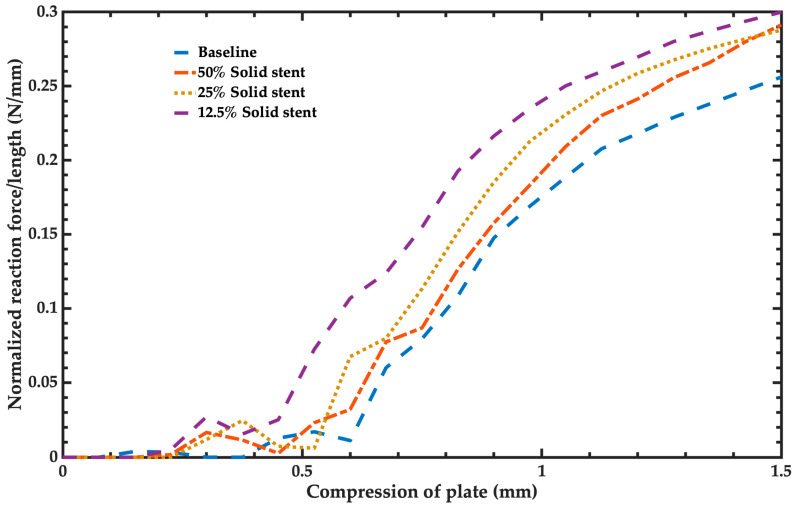
Normalized reaction force per length of the baseline model and the simplified solid models of the stent.

The radial stiffness of the stent was the third mechanical behavior parameter assessed using the simulation models. Figure 13 shows the normalized reaction force per length of the baseline model and the simplified solid models of the stents. The pattern is the same for all configurations; however, the model with behavior closest to the baseline model was the simplified solid model of 25% concerning the final normalized force. The second better simplified solid model was the 50% solid model. The third better solid model was the 12.5% solid model. The results of Figure 13 show a nonlinear behavior due to geometry buckling and material plasticity.

The simplified models of stents can predict their main mechanical performance parameters using a reduced computational time. Furthermore, these simplified models can be used to adjust the stents’ geometry and find the stent’s best geometry. Table 3 compares the simplified models in terms of simulation time, simulation reduction time, radial stiffness error, radial recoil index error, radial recoil error, foreshortening difference error, and error summation. These parameters are ordered from the best to the least favorable value based on radial stiffness error as the primary criterion, followed by error summation as a secondary consideration. These data are compared to the baseline model to determine the best-simplified stent models. Based on the above criterion, the best-simplified model is the 50% solid model that registers a lesser difference concerning the baseline model. The second-best simplified model is the 25% model, which has an even more significant time reduction (5.5% faster). Shell models, particularly those utilizing formulations 1 and 2 with integration points greater than one across the thickness, demonstrate better performance in decreasing the simulation time and achieving good agreement with radial and foreshortening measurements. However, for the compression analysis, shell models have poor agreement compared to the baseline model. The results suggest a potential pathway to reduce simulation time during the initial stages of defining a new stent geometry. Table 4 depicts the results of simulation times of different modeling techniques of stents reported in the literature. Our simplified stent model achieved the best simulation time reduction. Thus, the results of the proposed simplified models can estimate the main mechanical performance parameters of stents considering the FDA guidelines and using a significant computational time reduction. Specifically, for a reference simulation point, almost five points related to the mechanical performance parameters of a stent can be obtained. This advancement may improve personalized stent designs tailored to individual patient conditions.

We validated a baseline model of the stent using experimental data. In addition, we developed simplified FEM models of stents to predict their mechanical performance parameters regarding FDA guidelines, decreasing the simulation time close to 80%. The proposed models can be used to design future stents with specific mechanical performance parameters suitable for the requirements of each patient.

## 4. Conclusions

Considering the FDA guidelines, simplified models of stents to predict their main mechanical performance parameters were proposed. These parameters considered the foreshortening, radial recoil, radial recoil index, and radial stiffness of stents. The simplified models were based on the finite element method using the software ANSYS. In addition, the results of the simplified models were compared to those of a baseline model of the stent. This baseline model was validated using experimental data. The best simplified model used solid elements, reducing computational time close to 80%. In addition, the proposed models can be used to optimize the design of stents for patients with specific requirements.

Future research on simplified stent models will consider additional parameters such as stent dog boning, quantitative measures of circumferential stress on the arterial wall, and quantitative measurements of radial stress on the arterial wall for structural analysis. Furthermore, we will use the hyperelastic and plasticity models to simulate the behavior of the balloon and the stent.

## Figures and Tables

**Table 1:** Plastic properties of the CrCo-L605 used in the FEM models of the stents.

Point	Strain	Stress (MPa)
1	6.6730 × 10^−3^	1117
2	7.6940 × 10^−3^	1178
3	8.7150 × 10^−3^	1235
4	1.0256 × 10^−2^	1293
5	1.2835 × 10^−2^	1345
6	1.6444 × 10^−2^	1389
7	2.0562 × 10^−2^	1416
8	2.5945 × 10^−2^	1442
9	3.0791 × 10^−2^	1461
10	3.5871 × 10^−2^	1472
11	4.2940 × 10^−2^	1486
12	4.9211 × 10^−2^	1496
13	1.6077 × 10^−1^	1677

**Table 2:** Recoil and recoil index of the simulation models of the stents.

Simulation Models	Recoil Index	Recoil
Baseline	2.00	1.09
12.5% Solid stent	2.00	1.11
25% Sell 1 stent-IP3	2.02	1.17
25% Shell 1 stent-IP5	2.03	1.16
25% Shell 2 stent-IP3	2.05	1.14
25% Shell 2 stent-IP5	2.06	1.12
25% Solid stent	1.93	1.11
25% Shell 1 stent-IP2	2.09	1.10
25% Shell 2 stent-IP2	2.10	1.10
50% solid stent	1.89	1.23
25% Shell 16 stent-IP5	2.22	1.10
25% Shell 16 stent-IP7	2.23	1.06
25% Shell 16 stent-IP3	2.23	1.07
25% Shell 16 stent-IP6	2.24	1.06

**Table 3:** Comparison of the simulation time and errors of the main performance parameters of the simplified models of stents with respect to the baseline model.

Model	Simulation Time (min)	Time Reduction (%)	Radial Stiffness Error @ 0.75 mm Compression	Recoil Index Error	Recoil Error	Foreshortening Error Difference
Baseline	835.25	-	-	-	-	-
50% Solid	280.03	66.5%	11.70%	5.5%	12.84%	3.40%
25% Solid	234.13	72.0%	16.46%	3.5%	1.83%	3.06%
12.5% Solid	216.70	74.1%	21.71%	0.0%	1.83%	3.67%
25% Shell 2-IP 5	187.67	77.4%	100.00%	3.0%	2.75%	2.80%
25% Shell 1-IP 5	192.05	77.0%	100.00%	1.5%	6.42%	1.87%
25% Shell 1-IP 2	189.20	77.6%	100.00%	4.5%	0.92%	6.78%
25% Shell 2-IP 2	185.17	77.5%	100.00%	5.0%	0.92%	6.71%
25% Shell 1-IP 3	188.92	77.3%	100.00%	1.0%	7.34%	4.76%
25% Shell 2-IP 3	187.00	77.8%	100.00%	2.5%	4.59%	6.56%
25% Shell 16-IP 7	151.60	82.4%	100.00%	11.5%	2.75%	1.47%
25% Shell 16-IP 3	144.68	81.8%	100.00%	11.5%	1.83%	5.10%
25% Shell 16-IP 5	146.93	82.7%	100.00%	11.0%	0.92%	10.40%
25% Shell 16-IP 6	149.38	82.1%	100.00%	12.0%	2.75%	8.24%

**Table 4:** Computer time used in different modeling techniques of stents.

Reference	Number of Computer Cores	Computer Time per Point Evaluation (h)	Software
Our work	14	3.6	LS-DYNA Explicit (R11.1.0)
[19]	32	33.5	LS-DYNA Implicit (R10.1.0)
[16]	32	160	Abaqus Explicit (V 6.12)
[17]	8	26	Abaqus Explicit (V 6.9.2)
[29]	6	24	Abaqus Explicit (V 6.9.1)
[12]	8	>24	Abaqus Explicit (V 6.9.1)
[24]	4	120	Abaqus Explicit (V 6.9.1)
[10]	*	48	Abaqus Explicit

## Data Availability

Data are contained within the article.
